# Relationship between Neutrophil-to-Lymphocyte Ratio and Liver Fibrosis in Nonalcoholic Fatty Liver Disease Among Adults in the United States: Data from the National Health and Nutrition Examination Survey 2017-2018

**DOI:** 10.5152/tjg.2024.23231

**Published:** 2024-04-01

**Authors:** Yuanyuan Wang, Siqi Guo, Yanfang He, Qiang Zhang, Ni Zhou, Da Wang, Ping Mai

**Affiliations:** 1 Jiangsu University School of Medicine, Zhenjiang, China; 2 First Clinical Medical College of Gansu University of Chinese Medicine, Lanzhou, China; 3 Department of Gastroenterology, Gansu Provincial People’s Hospital, Lanzhou, China

**Keywords:** Liver fibrosis, neutrophil-to-lymphocyte ratio, nonalcoholic fatty liver disease, vibration-controlled transient elastography

## Abstract

**Background/Aims:**

The relationship between neutrophil-to-lymphocyte ratio (NLR) and liver fibrosis in nonalcoholic fatty liver disease remains controversial. The aim of this study was to examine the association between NLR and liver fibrosis.

**Materials and Methods:**

We conducted a cross-sectional analysis using the National Health and Nutrition Examination Survey. Vibration-controlled transient elastography was used to assess liver fibrosis and its severity. Neutrophil-to-lymphocyte ratio was calculated as the ratio of neutrophil count to lymphocyte count.

**Results:**

This study included 1620 US adults with a mean age of 52.9 years, of which 53.3% were male. The obese population accounted for 62.5%, 68.5% had hypertension, 31.1% had diabetes, and 16% had significant liver fibrosis. After adjusting for all covariates, a positive correlation was observed between NLR and the severity of liver fibrosis (*β* = 0.57, 95% CI = 0.22-0.92, *P* = .001), which remained stable across different subgroups.

**Conclusion:**

This study suggests that elevated NLR levels are positively correlated with the severity of liver fibrosis in patients with nonalcoholic fatty liver disease, and these results can be well generalized to the US adult population.

**Main Points**

- There is controversy regarding the relationship between neutrophil-to-lymphocyte ratio (NLR) and liver fibrosis.
- We have found that higher NLR levels are positively correlated with the severity of liver fibrosis.
- This result can be well generalized to the adult population in the United States.

## Introduction

Nonalcoholic fatty liver disease (NAFLD) is a metabolic disorder-related liver disease characterized by excessive fat deposition in the liver, independent of alcohol consumption, or other features commonly associated with liver diseases.^1^ The prevalence of NAFLD is approximately 25% in Western countries and over 10% in Asian countries.^2^ It is anticipated that the prevalence of NAFLD will continue to rise in the future.^3^ Nonalcoholic fatty liver disease exhibits a strong association with metabolic syndrome, obesity, type 2 diabetes, liver cancer, bladder cancer, and various other disorders, hence imposing a significant cost on society.^4,5^ Therefore, the prevention and treatment of NAFLD are crucial public health issues. Recent literature has highlighted the development of numerous new drugs, natural products, and other treatments for NAFLD.^6,7^ A significant stage in the development of NAFLD is liver fibrosis, which is characterized by increased deposition and proliferation of fibrous tissue in the liver as a result of long-term fat deposition and inflammatory damage, leading to severe impairments in liver structure and function.^8 ^The advancement of liver fibrosis is closely associated with the severity of NAFLD and may also lead to the development of liver cirrhosis and liver cancer, posing a threat to the health and life of patients.^9^

The gold standard for diagnosing liver fibrosis and cirrhosis is liver biopsy, although it should be noted that this process is invasive, difficult, and associated with inherent risks and limits.^10^ Vibration-controlled transient elastography (VCTE), also known as FibroScan®, is a noninvasive and convenient examination method that has been widely used to assess the degree of liver fibrosis. It allows for rapid and accurate assessment of disease progression and prognosis in patients, with significant clinical relevance.^11^ The neutrophil-to-lymphocyte ratio (NLR) is an indicator that directly reflects the level of inflammation and has been extensively utilized in the diagnosis and prognosis evaluation of various diseases.^12-14^ Over the past several years, a substantial amount of research has emerged suggesting that the NLR can serve as a biomarker for evaluating the occurrence and intensity of liver fibrosis in individuals with NAFLD. Numerous studies have yielded inconclusive findings about the association between NLR and liver fibrosis.^15-17^

In conclusion, NLR holds potential as an indicator of inflammation in the diagnosis and prognosis evaluation of NAFLD and liver fibrosis. Nevertheless, there is still controversy and uncertainty regarding its correlation with liver fibrosis, necessitating further evidence.

## Materials and Methods

### Study Population

The National Health and Nutrition Examination Survey (NHANES) is a research program aimed at assessing the health and nutritional status of adults and children in the United States. The survey combines interviews and physical examinations, contributing to the development of sound public health policies, guiding and designing health programs and services, and expanding the nation’s health knowledge.^18^ All NHANES data are available for viewing and use on the Centers for Disease Control and Prevention (CDC) website (https://www.cdc.gov/nchs/nhanes). The National Center for Health Statistics Research Ethics Review Board reviewed and approved NHANES. The NHANES is a public-use dataset available through the website. In accordance with national legislation and institutional equirements, Ethics Committee Approval was not deemed necessary for this study. All participants provided signed informed permission.

**Figure 1.**
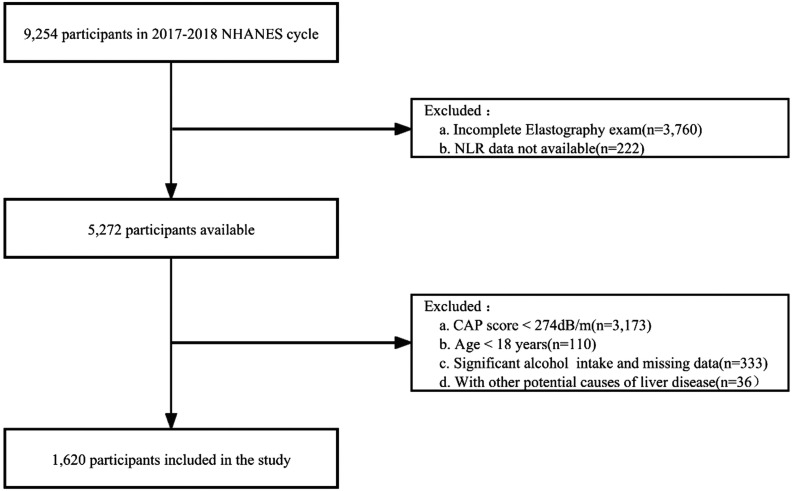
Flowchart of study. CAP, controlled attenuation parameter; NHANES, National Health and Nutrition Examination Survey; NLR, neutrophil-to-lymphocyte ratio.

We used the 2017-2018 NHANES data for our cross-sectional study, which included a sample of 9254 individuals from 1 survey cycle. The FibroScan® device, using VCTE, was utilized to measure liver stiffness, which is an indicator of liver fibrosis. The device also measured controlled attenuation parameter (CAP), which is an ultrasound attenuation index related to liver fat. Based on previous studies, we defined CAP ≥ 274 dB/m as indicating the presence of NAFLD.^19^ We excluded 3760 participants who did not complete the VCTE examination, as well as 222 participants with missing NLR data. Additionally, we excluded individuals who were not diagnosed with NAFLD, were under the age of 18, had a history of heavy alcohol consumption (≥30 g/day for males, ≥20 g/day for females), or had missing alcohol consumption data ^20^ Participants with other chronic liver diseases, such as viral hepatitis infection (positive for hepatitis B surface antigen, positive for hepatitis C antibody, or positive ribonucleic acid), autoimmune hepatitis, or liver cancer, were also excluded. Ultimately, a total of 1620 participants who met the inclusion and exclusion criteria for NAFLD were included in the analysis. The study flowchart is shown in Figure 1.

### Assessment of Liver Fibrosis

At the NHANES Mobile Examination Center (MEC), participants underwent examination with the FibroScan® device using a medium or extra-large probe to obtain CAP and liver stiffness data. Participants were required to undergo a complete examination, including a fasting period of at least 3 hours and a minimum of 10 valid measurements of liver stiffness with an interquartile range/median liver stiffness <30%, in order to be included in the study. Based on a prospective study involving NAFLD patients, a median liver stiffness of ≥8.2 kPa was considered indicative of significant fibrosis (≥F2).^19^

### Neutrophil-to-Lymphocyte Ratio

Complete blood count (CBC) is a routine blood test, and whole blood specimens were analyzed at the NHANES MEC using a Beckman Coulter DxH 800 instrument to generate CBC results for all participants. The laboratory methods used can be found in the NHANES Laboratory Methods file section (https://wwwn.cdc.gov/nchs/nhanes/). In this study, the NLR was calculated as the neutrophil count divided by the lymphocyte count, with both neutrophil and lymphocyte counts expressed as ×1000 cells/µL.

### Covariates

Based on clinical experience and previous literature,^15-17,21,22^ we selected age, race/ethnicity, sex, level of education, body mass index BMI (kg/m^2^), waist circumference (cm), physical activity, smoking status, aspartate aminotransferase (U/L), alanine transaminase (U/L), alkaline phosphatase (U/L), gamma glutamyl transferase (U/L), total bilirubin (µmol/L), triglyceride (mmol/L), hypertension, and diabetes mellitus as covariates. The race/ethnicity categories were Mexican American, other Hispanic, nonHispanic White, nonHispanic Black, and other race. There were 3 education categories: below high school, high school, and beyond high school. Body mass index was classified as under/normal weight, overweight, or obese (25, 25-30, 30). Physical activity was categorized as vigorous, moderate, or inactive based on the answers to the following questions: “Do you do any vigorous intensity exercise, fitness, or recreational activity during the week?” and “Do you do any moderate intensity exercise, fitness, or recreational activity that causes a slight increase in breathing or heart rate during the week?” There were 3 types of smoking status: Non-smokers are individuals who never used to smoke or smoked no more than 100 cigarettes in their lifetime, former smokers are adults who have smoked more than 100 cigarettes in a lifetime but have ceased smoking as of the interview, and current smokers are adults who are currently smoking. The triglyceride–glucose (TyG) index is represented using a logarithmic scale and is determined using the following formula: Ln [fasting triglycerides (mg/dL) × fasting glucose (mg/dL)/2]. People with hypertension were recognized in 1 of 2 ways: SBP > 130 mmHg or DBP > 80 mmHg, or are currently using high blood pressure (HBP) medication, or have been diagnosed with hypertension by a doctor or other health professional. Diabetics were defined as those with a glycosylated hemoglobin concentration of 6.5% or a fasting glucose concentration of more than 126 mg/dL, or those who answered yes to the questions “Did your doctor tell you that you have diabetes?” or “Are you currently taking insulin?”

### Statistical Analysis

When a continuous variable had a distribution that was normal, the means and standard errors were presented; when it had a skewed distribution, the median and interquartile spacing were expressed; and when it had a categorical distribution, the frequencies and percentages were expressed. Normality of all variables was assessed, and skewed variables were normalized by logarithmic transformation when appropriate. If a covariate altered the NLR estimate of liver fibrosis by over 10% or had a strong correlation with liver fibrosis in a univariate research (*P* < .1), it was included as a potential confounder in the final model. To examine the multicollinearity of all variables, variance inflation factors were used. Multivariate logistic regression models were used to investigate the relationship between NLR and liver fibrosis, whereas multivariate linear regression models were used to investigate the relationship between NLR and the severity of liver fibrosis. Model I only had minor sociodemographic variable changes. Model II considered every variable. In addition, we separated NLR into 3 equal subgroups to evaluate linear trend lines, and we utilized stratified multiple regression analysis to conduct interaction and subgroup analyses to uncover associations unique to each subgroup. For all analyses, the R software program version 4.2.0 was used, and *P* < .05 indicated statistical significance.

## Results

### Demographic Characteristics

Table 1 presents the baseline characteristics of the participants. The mean age of all participants was 52.9 ± 16.7 years, with 863 males (53.3%), 579 non-Hispanic White individuals (35.7%), 1260 individuals with a high school education or above (79.8%), 1007 obese individuals (62.5%), 1110 individuals with hypertension (68.5%), 507 individuals with diabetes (31.1%), and an average NLR of 2.1 ± 1.1.

### Relationship Between Neutrophil-to-Lymphocyte Ratio and Liver Fibrosis

Table 2 depicts the connection between NLR and liver fibrosis. When NLR was treated as a continuous variable, in the unadjusted model (odds ratio (OR) = 1.15, 95% CI = 1.03-1.29, *P* = .012), each unit increase in NLR was associated with a 15% increased risk of liver fibrosis. In Model I, adjusting for age, sex, and race, a weak similar association was still detected (OR = 1.12, 95% CI = 1.00-1.26, *P *= .049). However, after adjusting for all covariates, there was no correlation between NLR as a continuous variable and liver fibrosis (OR = 1.01, 95% CI = 0.82-1.24, *P* = .914). When NLR was categorized into tertiles, similar results were observed. In the unadjusted model, compared to the Q1 group, there was a correlation between NLR in the Q3 group and liver fibrosis (OR = 1.55, 95% CI = 1.12-2.15, *P* = .009), while no correlation was found in the Q2 group. After adjusting all coordinates, it was shown that there was no link between the NLR and liver fibrosis.

### Relationship Between Neutrophil-to-Lymphocyte Ratio and Severity of Liver Fibrosis

Table 3 demonstrates the relationship between NLR and severity of liver fibrosis. In the unadjusted model (*β* = 0.52, 95% CI = 0.27-0.77, *P* < .001), Model I (*β* = 0.49, 95% CI = 0.24-0.75, *P* < .001), and Model II (*β* = 0.32, 95% CI = 0.06-0.57, *P *< .014), a clear positive correlation between NLR and the severity of liver fibrosis was observed. When NLR was categorized into tertiles (Q1, Q2, Q3), it was found that the Q3 group had a significant positive correlation with the severity of liver fibrosis compared to the Q1 group (*β* = 0.93, 95% CI = 0.03-1.84, *P* = .044).

### Subgroup Analysis

**Figure 2.**
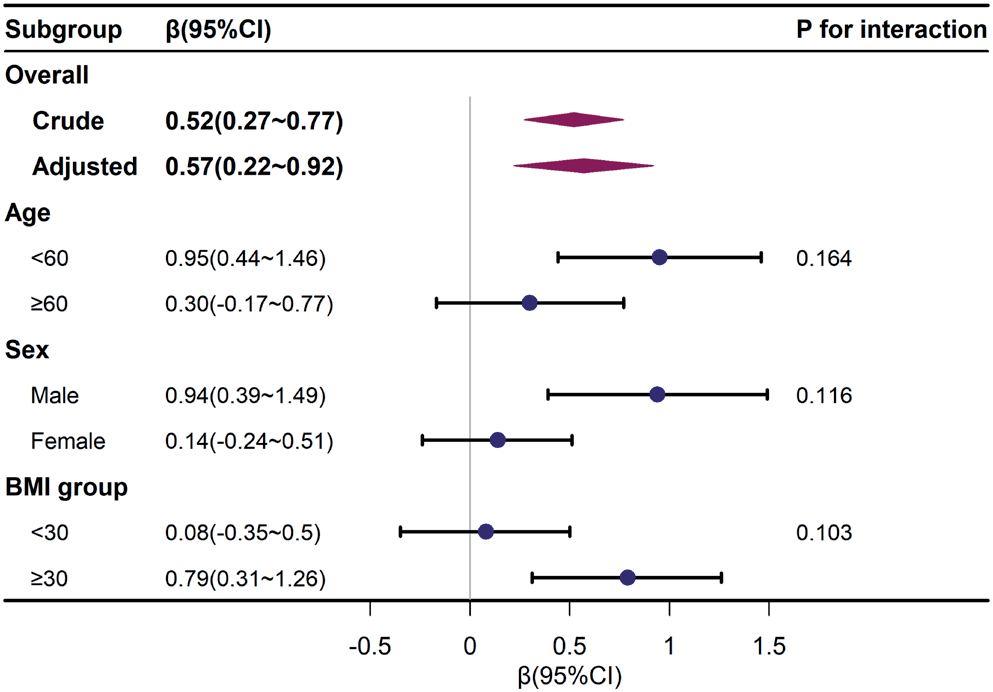
Subgroup analysis for neutrophil-to-lymphocyte ratio and severity of liver fibrosis. Adjusted for race, education level, physical activity, smoking status, alanine transaminase, aspartate aminotransferase, alkaline phosphatase, gamma glutamyl transferase, total bilirubin, triglyceride–glucose index, hypertension, and diabetes except the subgroup variable. BMI, body mass index.

**Figure 3.**
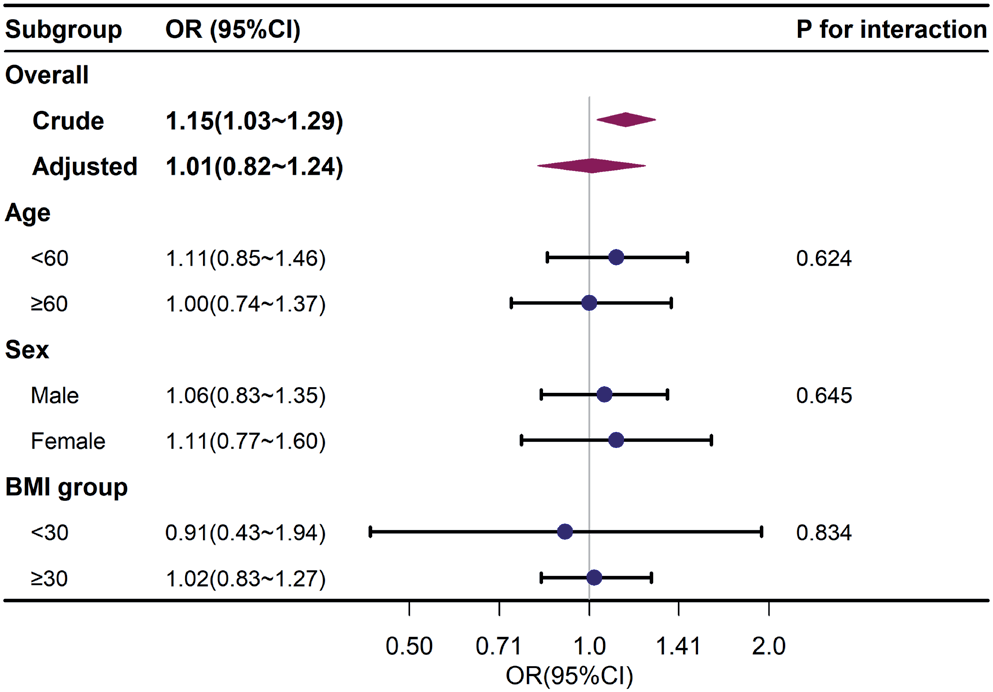
Subgroup analysis for neutrophil-to-lymphocyte ratio and liver fibrosis. Adjusted for age, sex, race, education level, body mass index, physical activity, smoking status, alanine transaminase, aspartate aminotransferase, alkaline phosphatase, gamma glutamyl transferase, total bilirubin, triglyceride, hypertension, and diabetes except the subgroup variable. BMI, body mass index. OR, odds ratio.

To further explore the influence of NLR on liver fibrosis and its severity, a stratified analysis using logistic regression was conducted. These models adjusted for all variables except for the stratifying variable. The results in Figure 2 and Figure 3 showed that age, sex, and BMI did not significantly modify the associations between NLR and liver fibrosis or the severity of liver fibrosis, indicating the stability of this association across different subgroups (*P* for interaction >.05).

## Discussion

In this cross-sectional investigation of American adults, we observed a positive correlation between NLR and the severity of NAFLD liver fibrosis (*β* = 0.57, 95% CI = 0.22-0.92, *P* = .001). This correlation was observed in both the unadjusted and minimally adjusted models; however, after adjusting for all covariates, there was no significant association between NLR and liver fibrosis (OR = 1.01, 95% CI = 0.82-1.24, *P* = .914). Additionally, there was no significant variation observed in different groups (*P* for interaction > .05).

Previously, Yilmaz et al^16^ found a positive correlation between NLR and the severity of liver fibrosis (*β* = 0.631, *P* < .001) in 38 patients with nonalcoholic steatohepatitis (NASH) confirmed by liver biopsy, and it was found to be a better predictor of liver fibrosis in NASH patients than C-reactive protein. Similarly, in a cohort of 101 NAFLD patients who underwent liver biopsy, Alkhouri et al^17^ discovered that for every unit increase in NLR, the risk of liver fibrosis increased by 50%. However, it should be noted that his sample size was limited to a university medical center and may not be representative of the general population. In addition to the liver biopsy, VCTE has provided similar evidence. Lesmana et al^22^ found a significant positive correlation between NLR and the severity of liver fibrosis in 106 NAFLD patients using TE examination. Our study obtained similar results. In contrast, WenYi et al^15^ reported a negative correlation between NLR and liver fibrosis (OR = 0.57, 95% CI = 0.35-0.94, *P* = .028) in a cohort of Chinese NAFLD patients confirmed by liver biopsy. However, this discrepancy may be attributed to the limited number of liver fibrosis patients in their study (n = 7). The inclusion of different populations, regions, races, sample sizes, differences in study designs, and the consideration of various confounding factors in these studies could have influenced the results.

The mechanism underlying in the relationship between NLR and liver fibrosis remains incompletely understood. However, several studies have proposed possible explanations. First, inflammation is recognized as a key driver of liver fibrosis, and NLR has been shown to be associated with the level of inflammation.^23-26^ Specifically, an excessive inflammatory response can induce liver cell damage and death, thereby promoting fibrosis development.^27^ Second, both neutrophils and lymphocytes are immune cells involved in the oxidative stress response,^28,29^ and there is evidence indicating that oxidative stress and chronic inflammation play a significant role in the progression of liver fibrosis.^30^ Nonetheless, these explanations remain subject to ongoing debate, and further research is needed to confirm the mechanistic link between NLR and liver fibrosis.

Our study possesses several notable strengths: 1) The data we analyzed derives from a nationally representative large-scale survey in the United States, bolstering the statistical credibility and reliability of our findings. Moreover, our study boasts the largest sample size among previous investigations, enhancing the generalizability of our results to the American population. 2) We meticulously gathered confounding factors from prior studies and incorporated insulin resistance data that was previously absent, adhering to recommendations for considering and controlling potential confounders. 3) Our study’s employment of stratification and interaction effect analysis allows for better utilization of the available data and facilitates the drawing of valid conclusions in distinct subgroups. However, certain limitations of our study should be acknowledged. First, the cross-sectional design inherently impedes the establishment of causal relationships. Second, we solely relied on a single inflammatory marker, NLR, to assess patients’ inflammatory status, whereas a more comprehensive assessment could have been achieved by measuring blood cell counts repeatedly. Finally, our study results specifically pertain to the American population and may not be extrapolated to other populations. Consequently, future research should encompass longitudinal studies with broad ethnic representation, utilizing repeated whole blood cell counts to assess patients’ inflammatory status.

In summary, our study findings indicate a positive correlation between elevated NLR and the severity of NAFLD liver fibrosis within the adult population of the United States. Furthermore, this correlation remains consistent across different subgroups. These results provide compelling evidence to further advance research in this field. However, additional research is warranted to validate and replicate our findings, as well as to investigate the underlying mechanisms driving this association.

## Figures and Tables

**Table 1.** Characteristics of Participants

Characteristic	Total (n = 1620)	Significant Fibrosis		*P*
		No (n = 1360)	Yes (n = 260)	
Age (years, mean ± SD)	52.9 ± 16.7	52.5 ± 16.9	54.9 ± 15.6	.033
Sex, n (%)				.119
Male	863 (53.3)	713 (52.4)	150 (57.7)	
Female	757 (46.7)	647 (47.6)	110 (42.3)	
Race/Ethnicity, n (%)				
Mexican American	320 (19.8)	265 (19.5)	55 (21.2)	.362
Other Hispanic	152 (9.4)	124 (9.1)	28 (10.8)	
Non-Hispanic White	579 (35.7)	479 (35.2)	100 (38.5)	
Non-Hispanic Black	279 (17.2)	240 (17.6)	39 (15.0)	
Other race	290 (17.9)	252 (18.5)	38 (14.6)	
Education Level, n (%)^a^				
Below high school	154 (9.8)	124 (9.4)	30 (11.8)	.397
High school	164 (10.4)	141 (10.6)	23 (9.1)	
Above high school	1260 (79.8)	1059 (80.0)	201 (79.1)	
BMI Group, n (%)^b^				
<30	604 (37.5)	563 (41.7)	41(15.8)	<.001
≥30	1007 (62.5)	788 (58.3)	219 (84.2)	
Waist (cm, mean ± SD)^C^	109.3 ± 15.2	107.1 ± 13.9	121.5 ± 16.0	<.001
Physical Activity, n (%)				.018
Vigorous	296 (18.3)	264 (19.4)	32 (12.3)	
Moderate	372 (23.0)	313 (23.0)	59 (22.7)	
Inactive	952 (58.8)	783 (57.6)	169 (65.0)	
Smoking Status, n (%)				
Never	953 (58.8)	811 (59.6)	142 (54.6)	.062
Former	440 (27.2)	354 (26.0)	86 (33.1)	
Current	227 (14.0)	195 (14.3)	32 (12.3)	
ALT (U/L, mean ± SD)^d^	1.3 ± 0.2	1.3 ± 0.2	1.4 ± 0.3	<.001
AST (U/L, mean ± SD)^e^	1.3 ± 0.2	1.3 ± 0.1	1.4 ± 0.2	<.001
ALP (IU/L, mean ± SD)^f^	82.9 ± 25.0	82.1 ± 23.7	86.9 ± 30.3	.004
GGT (IU/L, mean ± SD)^g^	1.4 ± 0.3	1.4 ± 0.3	1.6 ± 0.3	<.001
TBil (µmol/L, mean ± SD)^h^	0.8 ± 0.2	0.8 ± 0.2	0.9 ± 0.2	.036
TyG index (mean ± SD)^i^	9.0 ± 0.7	9.0 ± 0.6	9.2 ± 0.8	<.001
Hypertension, n (%)				
Yes	1110 (68.5)	911 (67.0)	199 (76.5)	.002
No	510 (31.5)	449 (33.0)	61 (23.5)	
Diabetes, n (%)				
Yes	507 (31.3)	374 (27.5)	133 (51.2)	<.001
No	1113 (68.7)	986 (72.5)	127 (48.8)	
NLR (mean ± SD)	2.1 ± 1.1	2.0 ± 1.1	2.2 ± 1.1	.011

BMI, body mass index; ALP, alkaline phosphatase; ALT, alanine transaminase; AST, aspartate aminotransferase; GGT, gamma glutamyl transferase; NLR, neutrophil-to-lymphocyte ratio; TBil, total bilirubin; TyG, triglyceride–glucose.

^a^Excludes 42 participants for whom data on education level was not collected;

^b^Excludes 9 participants for whom data on BMI group was not collected.

^c^Excludes 39 participants for whom data on waist was not collected.

^d^Excludes 27 participants for whom data on ALT was not collected.

^e^Excludes 33 participants for whom data on AST was not collected.

^f^Excludes 27 participants for whom data on ALP was not collected.

^g^Excludes 27 participants for whom data on GGT was not collected.

^h^Excludes 26 participants for whom data on TBil was not collected.

^i^Excludes 848 participants for whom data on TyG Index was not collected.

**Table 2.** Relationship Between Neutrophil-to-Lymphocyte Ratio and Liver Fibrosis

Outcome	Crude Model		Model I		Model II	
	OR (95% CI)	*P*	OR (95% CI)	*P*	OR (95% CI)	*P*
NLR	1.15 (1.03-1.29)	.012	1.12 (1.00-1.26)	.049	1.01 (0.82-1.24)	.914
NLR (quartile)						
Q1	Reference		Reference		Reference	
Q2	1.20 (0.85-1.68)	.299	1.19 (0.84-1.68)	.327	1.04 (0.57-1.90)	.897
Q3	1.55 (1.12-2.15)	.009	1.47 (1.04-2.06)	.027	1.17 (0.65-2.09)	.600
*P* for trend	.008		.026		.590	

Model I adjusted for age, sex, race. Model II adjusted for age, sex, race, education level, body mass index, physical activity, smoking status, alanine transaminase, aspartate aminotransferase, alkaline phosphatase, gamma glutamyl transferase, total bilirubin, triglyceride–glucose index, hypertension, and diabetes.

NLR, neutrophil-to-lymphocyte ratio; OR, odds ratio.

**Table 3.** Relationship Between Neutrophil-to-Lymphocyte Ratio and Severity of Liver Fibrosis

Outcome	Crude Model		Model I		Model II	
	*β* (95% CI)	*P*	*β* (95% CI)	*P*	*β* (95% CI)	*P*
NLR	0.52 (0.27-0.77)	<.001	0.49 (0.24-0.75)	<.001	0.57 (0.22-0.92)	.001
NLR (quartile)						
Q1	Reference		Reference		Reference	
Q2	0.54 (−0.12-1.19)	.109	0.41 (−0.26-1.08)	.228	0.25 (−0.65-1.16)	.586
Q3	1.10 (0.44-1.75)	.001	0.96 (0.28-1.64)	.006	0.93 (0.03-1.84)	.044
*P* for trend	.001		.005		.043	

Model I adjusted for age, sex, race. Model II adjusted for age, sex, race, education level, body mass index, physical activity, smoking status, alanine transaminase, aspartate aminotransferase, alkaline phosphatase, gamma glutamyl transferase, total bilirubin, triglyceride–glucose index, hypertension, and diabetes.

NLR, neutrophil-to-lymphocyte ratio.
